# Fiberoptic endoscopic evaluation of swallowing in early-to-advanced stage Huntington’s disease

**DOI:** 10.1038/s41598-020-72250-w

**Published:** 2020-09-17

**Authors:** Antonio Schindler, Nicole Pizzorni, Jenny Sassone, Lorenzo Nanetti, Anna Castaldo, Barbara Poletti, Federica Solca, Francesca Pirola, Laura Lazzari, Marco Stramba-Badiale, Agnese Rossi, Vincenzo Silani, Caterina Mariotti, Andrea Ciammola

**Affiliations:** 1 Phoniatric Unit, Department of Biomedical and Clinical Sciences “L. Sacco”, University of Milan, Via GB Grassi 74, 20157 Milan, Italy; 2 Vita-Salute San Raffaele University, Milan, Italy; 3 Division of Neuroscience, San Raffaele Scientific Institute, Milan, Italy; 4 Unit of Medical Genetics and Neurogenetics, Department of Diagnostics and Technology, Fondazione IRCCS Istituto Neurologico Carlo Besta, Milan, Italy; 5 Department of Neurology and Laboratory of Neuroscience, Istituto Auxologico Italiano IRCCS, P.le Brescia 20, 20149 Milan, Italy; 6 Department of Geriatrics and Cardiovascular Medicine, Istituto Auxologico Italiano IRCCS, Milan, Italy; 7 Department of Pathophysiology and Transplantation, Dino Ferrari Center, Università degli Studi di Milano, Milan, Italy; 8 Aldo Ravelli Center for Neurotechnology and Experimental Brain Therapeutics, Università degli Studi di Milano, Milan, Italy

**Keywords:** Huntington's disease, Neurological disorders

## Abstract

Huntington's disease (HD) is a neurodegenerative disorder characterized by motor disturbances, cognitive decline, and behaviour changes. A well-recognized feature of advanced HD is dysphagia, which leads to malnutrition and aspiration pneumonia, the latter being the primary cause of death in HD. Previous studies have underscored the importance of dysphagia in HD patients with moderate-to-advanced stage disease, but it is unclear whether dysphagia affects patients already at an early stage of disease and whether genetic or clinical factors can predict its severity. We performed fiberoptic endoscopic evaluation of swallowing (FEES) in 61 patients with various stages of HD. Dysphagia was found in 35% of early-stage, 94% of moderate-stage, and 100% of advanced-stage HD. Silent aspiration was found in 7.7% of early-stage, 11.8% of moderate-stage, and 27.8% of advanced-stage HD. A strong correlation was observed between disease progression and dysphagia severity: worse dysphagia was associated with worsening of motor symptoms. Dysphagia severity as assessed by FEES correlated with Huntington’s Disease Dysphagia Scale scores (a self-report questionnaire specific for evaluating swallowing in HD). The present findings add to our understanding of dysphagia onset and progression in HD. A better understanding of dysphagia onset and progression in HD may inform guidelines for standard clinical care in dysphagia, its recognition, and management.

## Introduction

Huntington's disease (HD) is an autosomal dominant neurodegenerative disorder caused by a CAG expansion in the *IT-15* gene; its prevalence in the Caucasian population is 7–11 per 100,000 (OMIM#143100). HD is characterized by motor, cognitive, and behavioural symptoms that have their onset usually between age 30 and 50 years, after which they slowly progress for 15–20 years until death. Most HD patients with moderate-to-advanced stages complain of swallowing difficulties. Severe dysphagia often leads to aspiration pneumonia, the main cause of death in HD^1^. The natural history of dysphagia in HD remains unclear.

Neuropathological changes in HD include prominent loss of striatal GABAergic neurons and progressive involvement of the cerebral cortex, pallidum, thalamus, brainstem, and cerebellum^2^. Such widespread neurodegeneration results in movement disorders. Besides chorea, the hallmark motor symptom in HD, other typical motor disorders include dystonia, incoordination, Parkinsonism, and ideomotor apraxia. When these heterogeneous movement disorders involve the oropharyngeal musculature, swallowing difficulties ensue^3^.

Dysphagia in HD has been investigated by subjective and objective swallowing evaluation tests and described in case reports and case series^1,3–6^. The Huntington’s Disease Dysphagia Scale (HDDS), a self-report questionnaire specifically designed to assess swallowing in HD, has demonstrated good construct. In patients with cognitive decline, however, it needs to be completed by carers who have a quite different perception of the patient’s swallowing difficulties than the patients themselves^7^. This is why integrating subjective swallowing evaluation with objective instrumental assessment is so important. In the largest study to date, dysphagia was objectively evaluated by means of videofluoroscopic swallowing study (VFSS) in a cohort of 35 HD patients with moderate-to-advanced stage disease^8^. More recently, fiberoptic endoscopic evaluation of swallowing (FEES) was described in small patient cohorts^6,9^. The studies underscored the importance of dysphagia in HD patients with moderate-to-advanced stage disease, but it remains unclear whether dysphagia affects patients at an early stage and when penetration/aspiration is likely to occur in HD progression. Moreover, what is also unclear is whether genetic or clinical factors can be used to predict the severity of dysphagia.

For this study, we used FEES to investigate dysphagia in a cohort of 61 HD patients with early-to-advanced disease and in a control group of 31 healthy subjects.

## Results

### Dysphagia assessment in controls and HD patients

**Figure 1:**
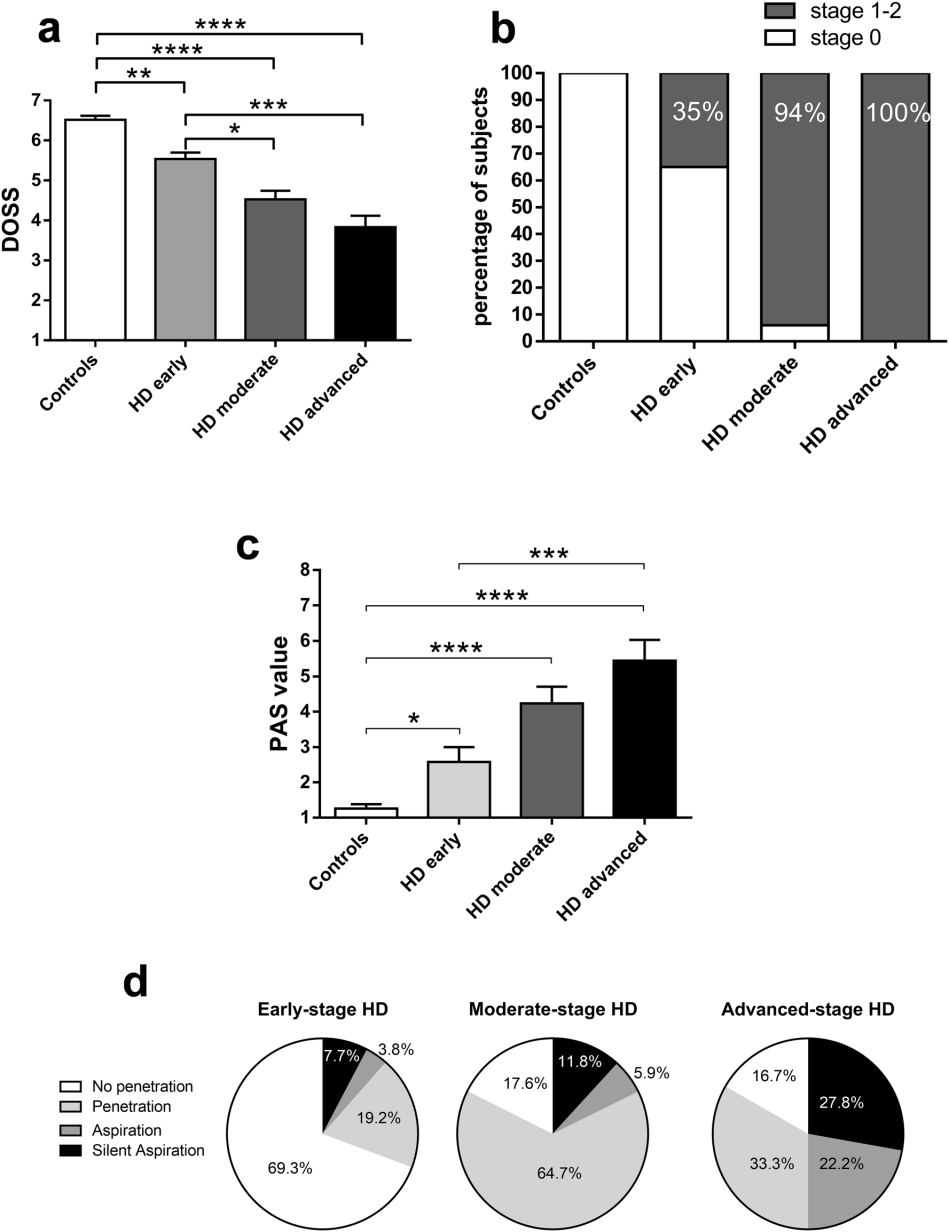
Dysphagia parameters in controls (n = 31) and HD patients (n = 61): early (n = 26), moderate (n = 17), and advanced HD (n = 18). (**a**) Comparison of the Dysphagia Outcome and Severity Scale (DOSS) level in HD patients and controls. DOSS levels were lower in early-moderate-advanced HD patients compared to controls (mean ± SEM of DOSS: controls 6.52 ± 0.10, early-stage 5.54 ± 0.16, moderate-stage 4.53 ± 0.21, advanced-stage HD 3.83 ± 0.28, Kruskal–Wallis and Dunn's test, *p < 0.05; **p < 0.01, ***p < 0.001, ****p < 0.0001). (**b**) Distribution of dysphagia frequency. Dysphagia was present in 35% of early-stage, 94% of moderate-stage, and 100% of advanced-stage HD patients (*p* < 0.0001, df 3, Chi-square 64.58). (**c**) Comparison of Penetration-Aspiration Scale (PAS) scores. PAS scores were higher for HD patients than for controls (mean ± SEM of PAS scores: controls 1.26 ± 0.12, early-stage 2.58 ± 0.42, moderate-stage 4.24 ± 0.47, advanced-stage HD patients 5.44 ± 0.58. Kruskal–Wallis and Dunn's test, *p < 0.05; ***p < 0.001, ****p < 0.0001). (**d**) Pie chart showing the percentage of silent aspiration, aspiration and penetration in HD patients.

Dysphagia severity was evaluated by FEES and graded according to the Dysphagia Outcome and Severity Scale (DOSS)^10^. The DOSS describes the overall severity of dysphagia based on signs of dysphagia detected during instrumental evaluation of swallowing (i.e., penetration, aspiration, and pharyngeal residue) and the ability of the patient to manage such signs. The score ranges from 7 to 1, with levels 7 and 6 indicating swallowing within functional limits, levels 5 to 3 indicating the presence of dysphagia, and levels 2 to 1 indicating severe swallowing impairment. FEES was well tolerated by all participants. As expected, DOSS scores were lower for the HD patients than the controls (HD 4.75 ± 0.15 vs. controls 6.52 ± 0.10; Mann–Whitney test, *p* < 0.0001). To determine whether dysphagia was present in the patients with early-stage HD, we stratified patients according to disease stage^11^ and reanalysed the DOSS scores. Despite the small study sample, the DOSS score was lower for the HD patients in the early stage (n = 26) than for the controls (controls 6.52 ± 0.10, early-stage HD 5.54 ± 0.16, moderate-stage HD 4.53 ± 0.21, advanced-stage HD 3.83 ± 0.28, Kruskal–Wallis and Dunn's test, controls vs. early-stage HD *p* < 0.01; controls vs. moderate-stage HD *p* < 0.0001; controls vs. advanced-stage HD *p* < 0.0001; early-stage vs. moderate-stage HD *p* < 0.05; early-stage- vs. advanced-stage HD *p* < 0.001; Fig. 1a).

To better quantify the frequency of dysphagia at each HD stage, we analysed the number of patients with normal swallowing (DOSS score 7–6 denotes dysphagia severity stage 0) versus those with dysfunctional swallowing (DOSS score ≤ 5 denotes dysphagia severity stages 1–2). Dysphagia was present in 100% of advanced-stage, 94% of moderate-stage, and 35% of early-stage HD patients (*p* < 0.0001, df 3, chi-square 64.58; Fig. 1b).

To better characterize dysphagia and estimate the risk of severe complications such as aspiration pneumonia, swallowing safety was assessed according to the Penetration-aspiration scale (PAS) score. Consistent with previous results, the PAS scores for the HD patients were higher than for the controls and worsened with progressive disease stage (controls 1.26 ± 0.12, early-stage 2.58 ± 0.42, moderate-stage 4.24 ± 0.47, advanced-stage HD patients 5.44 ± 0.58. Kruskal–Wallis and Dunn's test, controls vs. early-stage *p* < 0.05; controls vs. moderate-stage *p* < 0.0001; controls vs. advanced-stage HD patients *p* < 0.0001; early-stage vs. advanced-stage HD patients *p* < 0.001; Fig. 1c). Silent aspiration was found in 7.7% of patients with early-stage, 11.8% of those with moderate-stage, and 27.8% of those with advanced-stage HD. Impaired swallowing safety was already present in early-stage HD; the risk of lower airway invasion increased with disease progression (Fig. 1d).

Pharyngeal residue, defined as pharyngeal food residue not entirely cleared by a swallow, was associated with the risk of malnutrition^12^. We tested pharyngeal residue by means of the Yale Pharyngeal Residue Severity Rating Scale. Pharyngeal residue was present in patients with early stage HD (Supplementary Fig. S1).

The correlation between the HDDS questionnaire^7^ score and the DOSS level (Spearman, r = − 0.30, *p* = 0.024) showed that objectively determined dysphagia severity correlates with subjective self-report of dysphagia by the HD patients or their caregivers (Supplementary Fig. S2).

### Correlations between dysphagia and HD features

We wanted to determine whether genetic or clinical factors could be useful for predicting dysphagia severity. DOSS scores did not correlate with age and CAG values (Spearman, *p* > 0.05), however, they did correlate with duration of illness and disease burden^13^ (Spearman, duration of illness r = − 0.33, *p* = 0.0102, disease burden r = − 0.34, *p* = 0.0007; Supplementary Figs. S3, S4). We found a strong correlation between total functional capacity (TFC-UHDRS VI) and DOSS scores (Spearman, r = 0.58, *p* < 0.0001; Supplementary Fig. S5). A positive correlation was also found between UHDRS Parts IV-V and DOSS scores (Spearman, r = 0.51, *p* = 0.0001 for UHDRS IV and r = 0.54, *p* < 0.0001 for UHDRS V), indicating a correlation between disease progression and dysphagia severity.

**Figure 2:**
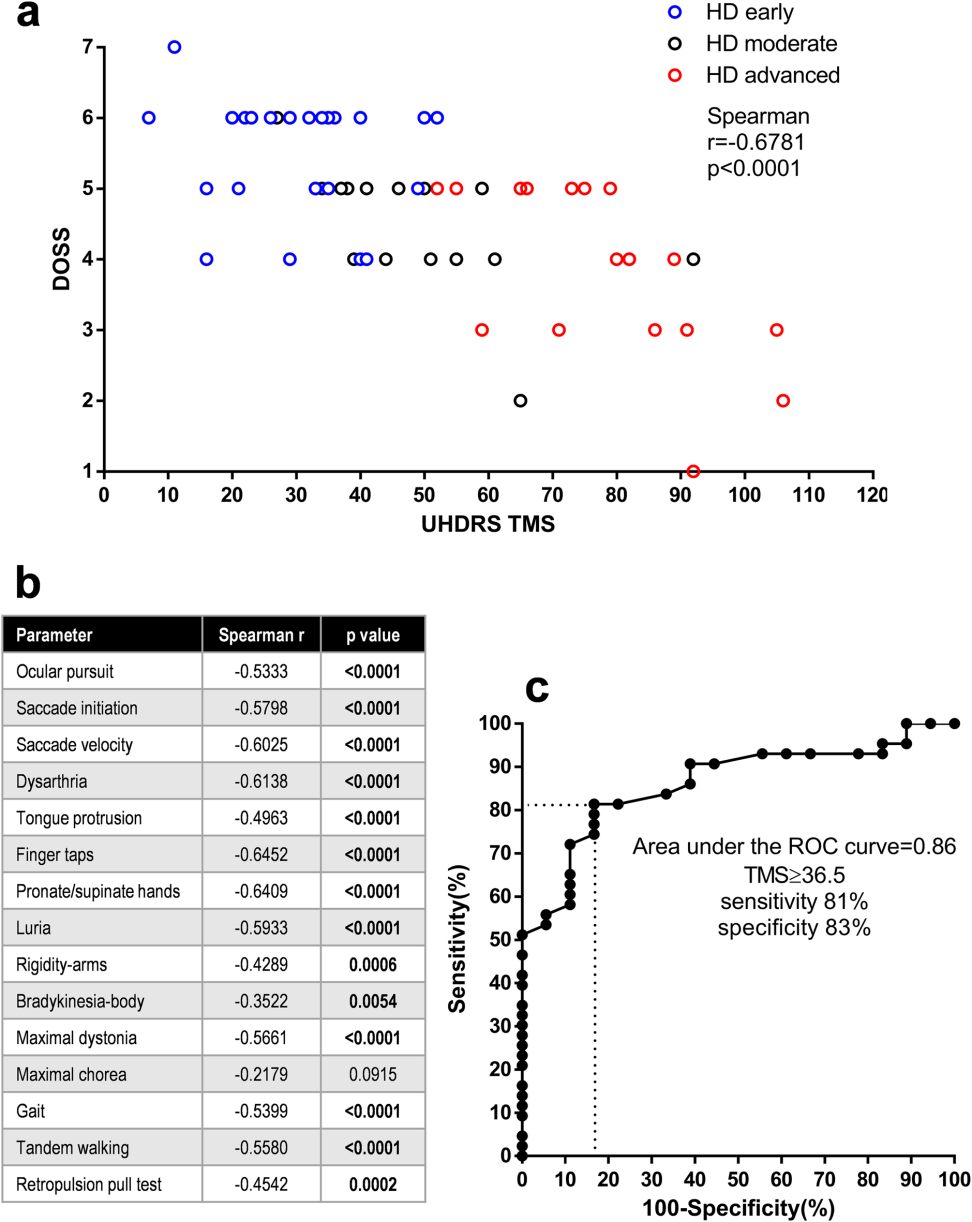
Correlations between dysphagia severity parameters and disease progression. (**a**) Correlation between DOSS levels and the total motor score in HD patients. A negative correlation was found between the two parameters. Spearman correlation coefficient r = − 0.6781, *p* < 0.0001. (**b**) Correlations between DOSS levels and UHDRS I subitem scores. All parameters but maximal chorea correlated negatively with DOSS level. **c** Receiver operating curve (ROC) of gain-of-function mutations and control mutations as a function of UHDRS I total motor score (TMS). Based on a cut-off TMS of 36.5 that maximizes sensitivity and specificity, TMS correctly classified 15 out of 18 HD patients as having normal swallowing (DOSS ≥ 6) and 35 out of 43 as having dysfunctional swallowing DOSS ≤ 5 (81% sensitivity and 83% specificity). The area under the curve is 0.86 (95% confidence interval = 0.77 to 0.95).

Because dysphagia arises when motor disturbances involve the oropharyngeal musculature, we hypothesized that dysphagia would worsen with worsening of motor symptoms. We found a strong negative correlation between total motor score (TMS) and DOSS score (Spearman, r = − 0.68, *p* < 0.0001; Fig. 2a). Also, all the UHDRS-TMS subitems, except maximal chorea, correlated with DOSS scores, suggesting that the heterogeneous movement disorders that characterize HD may contribute to the development of dysphagia (Fig. 2b).

Moreover, we hypothesized that the TMS might provide enough sensitivity and specificity to distinguish between HD patients with normal swallowing and those with dysfunctional swallowing. Using a cut-off of TMS 36.5, which maximizes sensitivity and specificity, we were able to correctly classify 15 out of 18 HD patients with normal swallowing (DOSS score ≥ 6) and 35 out of 43 of HD patients with dysfunctional swallowing (DOSS score ≤ 5) (81% sensitivity and 83% specificity) (Fig. 2c). Despite the small sample study, this result suggests that a motor impairment with a TMS > 36.5 could be predictive of dysphagia onset. Further studies in a larger cohort of patients are desirable to confirm this cut-off value.

## Discussion

Dysphagia severely reduces the quality of life of HD patients and their caregivers; it is associated with an increased risk for aspiration pneumonia, the primary cause of death in HD. Accurate swallowing assessment is essential for correct management of HD patients. FEES is a validated and widely used technique to assess the pharyngeal phase of swallowing^14^. FESS directly visualizes the anatomy of the pharynx and larynx. Although it does not provide real-time information on the oral phase of swallowing, it could be equal or even more sensitive than VFSS to identify tracheal aspiration and post-swallow residue^15,16^, which are closely correlated with increased risk of aspiration pneumonia.

This is the first study based on FEES to describe swallowing alterations in a relevant percentage (35%) of HD patients with early-stage disease. The objective demonstration of impaired swallowing in early HD stages is novel among studies on dysphagia in HD and shares evidence with a previous study that reported, via questionnaire, subjective swallowing difficulties very early in HD^17^. We further demonstrated that dysphagia worsens with disease progression and that dysphagia is frequent in patients with moderate- and advanced-stage HD. Analysis of the rate of penetration/aspiration and post swallowing residue showed that 7% of early-stage HD patients in this cohort displayed silent aspiration, which rose to 12% and 28% in those with moderate and advanced stage disease, respectively.

These findings show that abnormal swallowing can be detected by FEES already in early-stage HD patients. In this perspective, assessment with comprehensive subjective evaluation scales that can predict penetration or aspiration in HD patients takes on increased importance. Based on responses to a recently developed HD dysphagia-specific self-report questionnaire (HDDS)^7^, our data highlight a correlation between HDDS score and DOSS level; this result confirms the validity of the HDDS for the subjective assessment of dysphagia severity. Further studies involving larger cohorts are needed to strengthen the utility of the HDDS, or similar clinical evaluation scales, to predict the onset of dysphagia and associated serious complications such as tracheal aspiration.

Swallowing is a highly integrated and complex sensorimotor process^18^ that relies on neuromuscular coordination, adequate strength, precision, timing, speed of reaction, and planning of motor movements^19^. Dysphagia may result from the impairment of various motor and sensory components. We speculate that dysphagia in HD stems from three different mechanisms.

Our results show that HD patients display penetration/aspiration starting from an early-stage of disease. Altered coordination can lead to penetration-aspiration because of inadequate timing of swallowing events (poor oral control, delayed pharyngeal response, uncoordinated laryngeal closure). Penetration and aspiration may also occur before swallowing due to poor oral control and delayed pharyngeal response, during swallowing because of ineffective or uncoordinated protective mechanisms, or after swallowing secondary to pharyngeal residue^20^. Such neuromuscular discoordination, also named oropharyngeal dyssynergia, may stem from basal ganglia and cerebellar dysfunction^2^ and may be the main pathophysiological mechanism underlying dysphagia in HD patients^8,9^. Poor coordination of the oropharyngeal muscles can also lead to the food pharyngeal residue^9^ observed in HD starting from the early stages of disease. Another pathophysiological mechanism potentially underlying food pharyngeal residue is weakness of the oro-pharyngeal muscles. This can lead to ineffective propulsion of the bolus during swallowing^21,22^. Because mutant huntingtin is ubiquitously expressed in human tissues including muscle cells^23^, and because peripheral tissues of HD patients bear abnormalities related to the expression of mutant huntingtin^24^, we speculate that the oro-pharyngeal muscles are primarily affected by mutant huntingtin. Finally, the increased rate of silent aspiration in HD patients may result from sensory impairment^25^ of the epiglottis and posterior wall of the hypopharynx, as previously described in other neurodegenerative conditions such as Parkinson’s disease^26^. However, it remains to be determined whether sensory component of coughing is impaired in HD patients.

Summarizing, the pathophysiology of swallowing in HD probably reflects the complex pattern of neurodegeneration of the HD brain which, in addition to affecting the striatum, also involves areas of the cerebral cortex, thalamus, pallidum, brainstem and cerebellum^2^. This is probably the reason why DOSS levels correlated with 14 out of 15 UHDRS Part I sub-items. The absence of a correlation between DOSS level and maximal chorea score likely reflects the well-known reduction in chorea described in advanced stages of HD. Noteworthy, dysphagia severity, as assessed by DOSS, strongly correlated with the TMS and a TMS > 36.5 predicted swallowing dysfunction (81% sensitivity and 83% specificity). Further studies in larger cohorts are warranted to substantiate this finding.

Overall, our findings fill a knowledge gap by systematically examining dysphagia through various stages of HD^4^. Our data suggest that monitoring swallowing in HD patients is warranted starting at an early stage of disease and that instrumental assessment with the FEES can be performed together with bedside evaluation to characterize progression of dysphagia.

Finally, the study contributes to the limited literature on swallowing impairment in movement disorders. The hypothesis is emerging that swallowing impairment manifests in the initial phase of diseases and requires prompt evaluation^27^.

## Methods

### Study population

The study sample was 61 HD patients (CAG ≥ 39) evaluated by neurologists with expertise in HD (A.C., C.M., and L.N.) and assessed according to the Unified Huntington's Disease Rating Scale (UHDRS)^11^. Based on UHDRS Part VI (Functional Capacity), HD was staged as early in 26 patients (score 13-7; Shoulson–Fahn stage 1–2), moderate in 17 (score 6-4; Shoulson–Fahn stage 3), and advanced in 18 (score 3-0; Shoulson–Fahn stage 4–5)^11^. Exclusion criteria were: use of enteral nutrition, history of head and neck cancer, other neurological diseases, self-reported or documented dysphagia prior to HD diagnosis.

For the control group, 31 age-matched healthy volunteers were recruited (Supplementary Fig. S6). Inclusion criteria were: age > 20 years, no medical history of voice, swallowing, gastroenterological, respiratory, neurologic, metabolic, hematologic or neoplastic disorders. All participants underwent evaluation by a phoniatrician and completed a medical history questionnaire to screen for potential comorbidities. Demographic, genetic, and clinical data are reported in Supplementary Table S1.

### Instrumental assessment of swallowing

All participants underwent fiberoptic endoscopic examination of swallowing (FEES) to objectively assess dysphagia by an experienced phoniatrician. FEES was conducted with liquids (3 trials × 5–10–20 cc of blue dyed water), semisolids (3 trials × 5–10–20 cc of pudding), and solids (2 trials × half cracker). Each FEES was video-recorded, de-identified, and assessed by two independent speech and language pathologists (SLPs) blinded to the patients’ clinical data. Inter-rater agreement calculated using the linear weighted kappa coefficient was substantial. Dysphagia severity, swallowing safety, and swallowing efficacy were rated with validated ordinal scales. Swallowing safety refers to the ability to transfer the bolus from the mouth to the stomach without penetration or aspiration into the lower airways, which is associated with respiratory complications; swallowing efficacy refers to the ability to transfer the bolus from the mouth to the stomach without post-swallow pharyngeal residue and is associated with nutritional complications^28,29^.

Dysphagia severity was assessed according to the Dysphagia Outcome and Severity Scale (DOSS)^10^. The seven DOSS levels, from 7 (normal swallowing) to 1 (severe dysphagia), are based on signs of dysphagia, need for diet modifications, and type of nutrition. Levels 7 and 6 correspond to swallowing within functional limits (dysphagia severity stage 0), levels 5 to 3 to mild-moderate dysphagia requiring diet modifications (dysphagia severity stage 1), and levels 2 and 1 to severe dysphagia requiring tube feeding (dysphagia severity stage 2).

For the safety analysis, laryngeal penetration and aspiration were assessed according to the Penetration-aspiration Scale (PAS)^30^. Scoring, from 1 (no penetration and aspiration) to 8 (silent aspiration), takes three variables into account: penetration or aspiration, level of airway invasion, and ability to eject substances from airways. The worst PAS score for each subject was entered in the statistical analyses. Pharyngeal residue, a measure of swallowing efficacy, was rated according to the Yale Pharyngeal Residue Severity Rating Scale (YALE)^31^. The scale provides two scores based on the amount of post-swallow residue in the valleculae and the pyriform sinuses. Scores range from 1 (no residue) to 5 (severe residue). The worst YALE score for each subject was entered in the statistical analyses. Along with FEES, patient-reported dysphagia was investigated by asking the patients to fill in the HDDS questionnaire. For HD patients unable to independently complete the HDDS, the questionnaire was administered orally by a SLP or to the patient’s carer.

### Statistical analysis

All data are presented as the mean ± SEM. The D'Agostino and Pearson omnibus normality test was used to test data for normality and the Brown-Forsythe test and Bartlett's test were used to verify the assumption of homogeneity of group variance. If the criteria for normality and equal variance were satisfied, the data were analyzed with analysis of variance (ANOVA) followed by Tukey or Dunnett's multiple comparisons test. Tukey test was used to compares every mean with every other mean and Dunnett’s test was used to compare every mean to the control subject mean. If the criteria for normality and equal variance were not satisfied, the data were analyzed using the Kruskal–Wallis test, followed by Dunn's multiple comparisons test. Data distribution was analyzed using the Chi-Square test. Correlations were assessed using the Spearman test because the considered variables were not normally distributed.

### Ethics approval

The study was carried out according to the Declaration of Helsinki and approved by the Institutional Review Board of the Luigi Sacco Hospital and the Ethics Committee of the Istituto Auxologico Italiano IRCCS. Written, informed consent was obtained from all participants or their caregivers.

## Supplementary information

Supplementary Figure 1.

Supplementary Figure 2.

Supplementary Figure 3.

Supplementary Figure 4.

Supplementary Figure 5

Supplementary Figure 6.

Supplementary Table 1.
