# Supplementary figures and images for: Fiberoptic endoscopic evaluation of swallowing in early-to-advanced stage Huntington’s disease
Source: Sci Rep. 2020 Sep 17;10:15242. doi: 10.1038/s41598-020-72250-w (PMC7499207; doi:10.1038/s41598-020-72250-w)

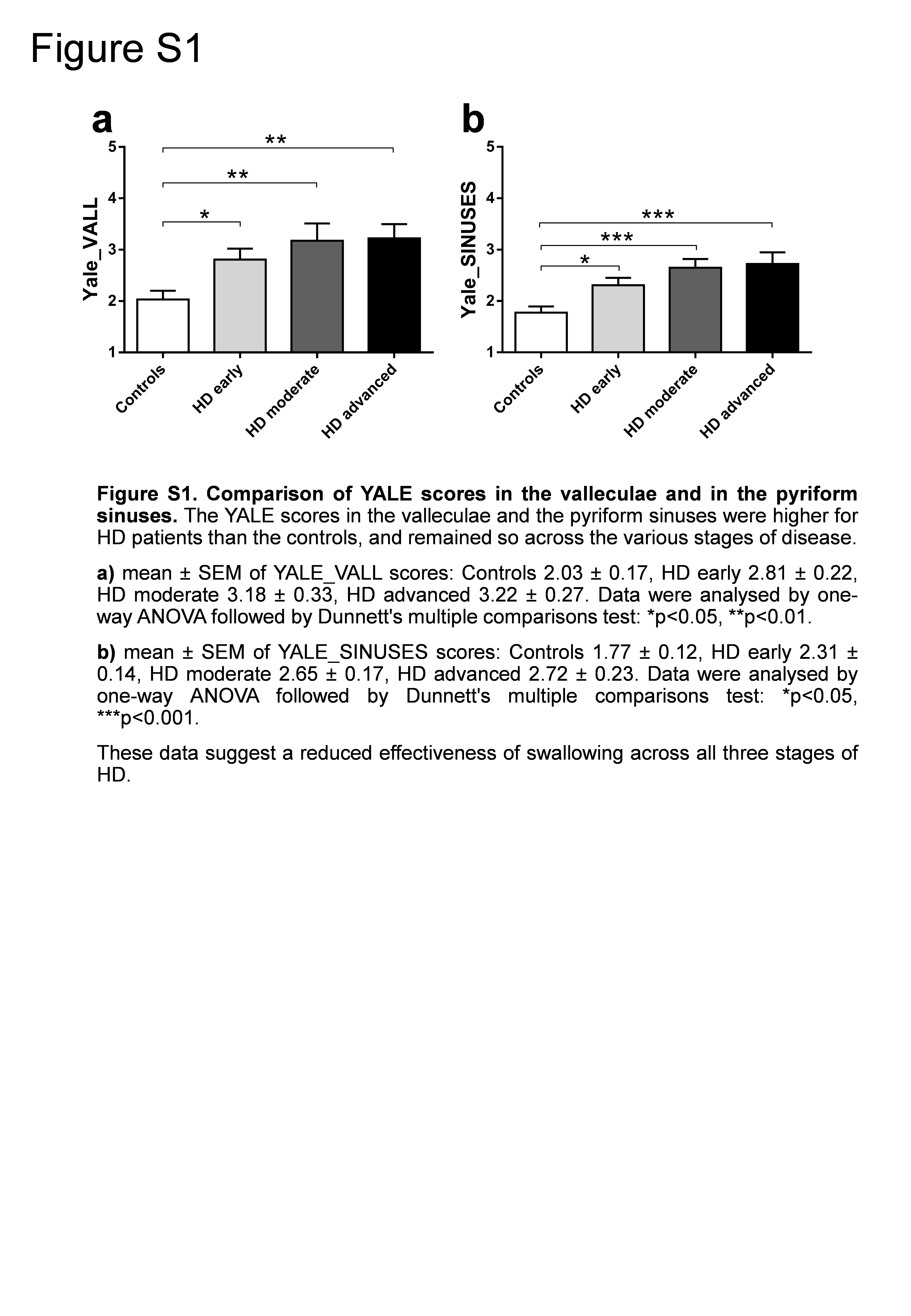

Supplement: Supplementary file 1 — Supplementary Figure 1. [file 41598_2020_72250_MOESM1_ESM.tif]

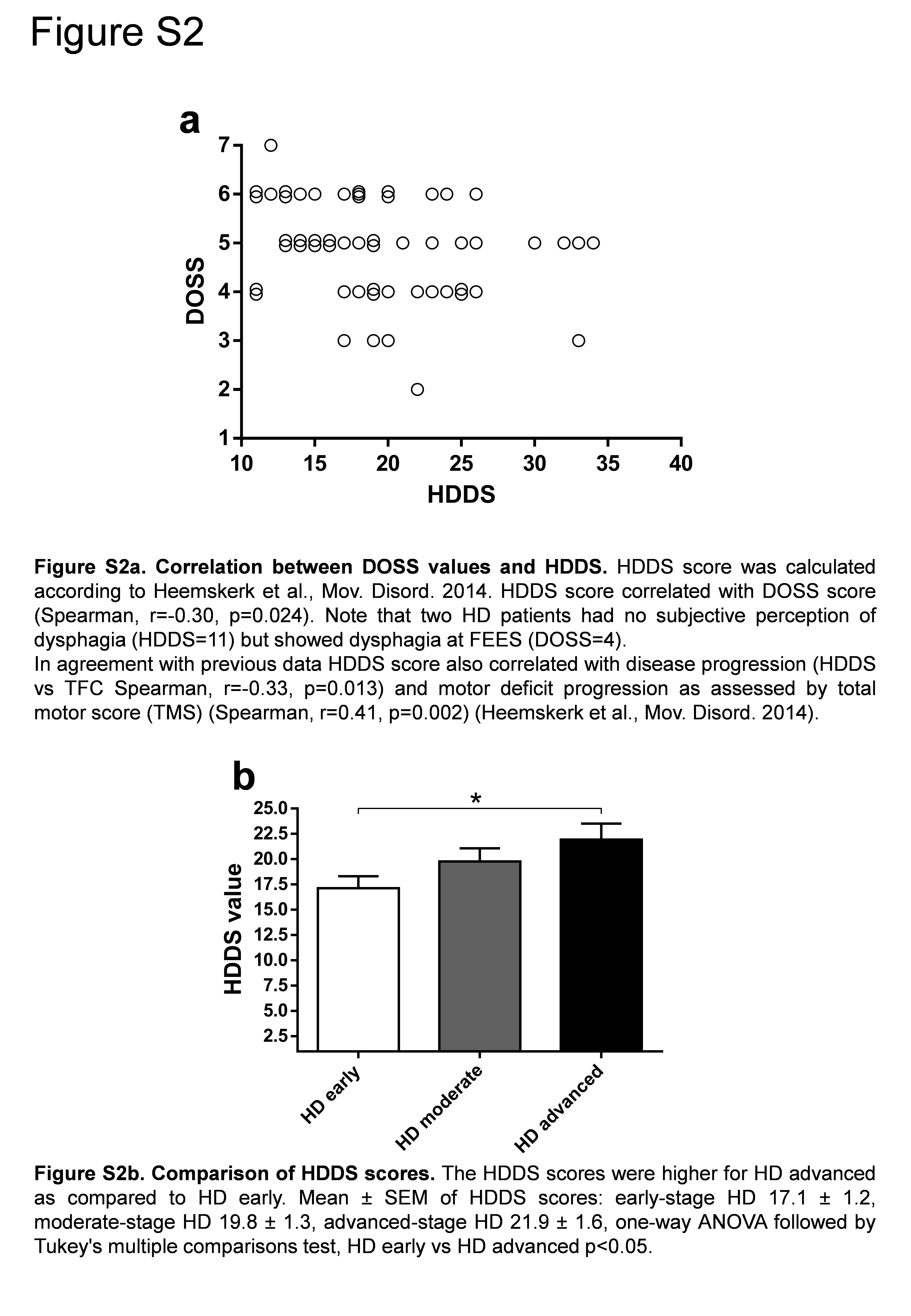

Supplement: Supplementary file 2 — Supplementary Figure 2. [file 41598_2020_72250_MOESM2_ESM.tif]

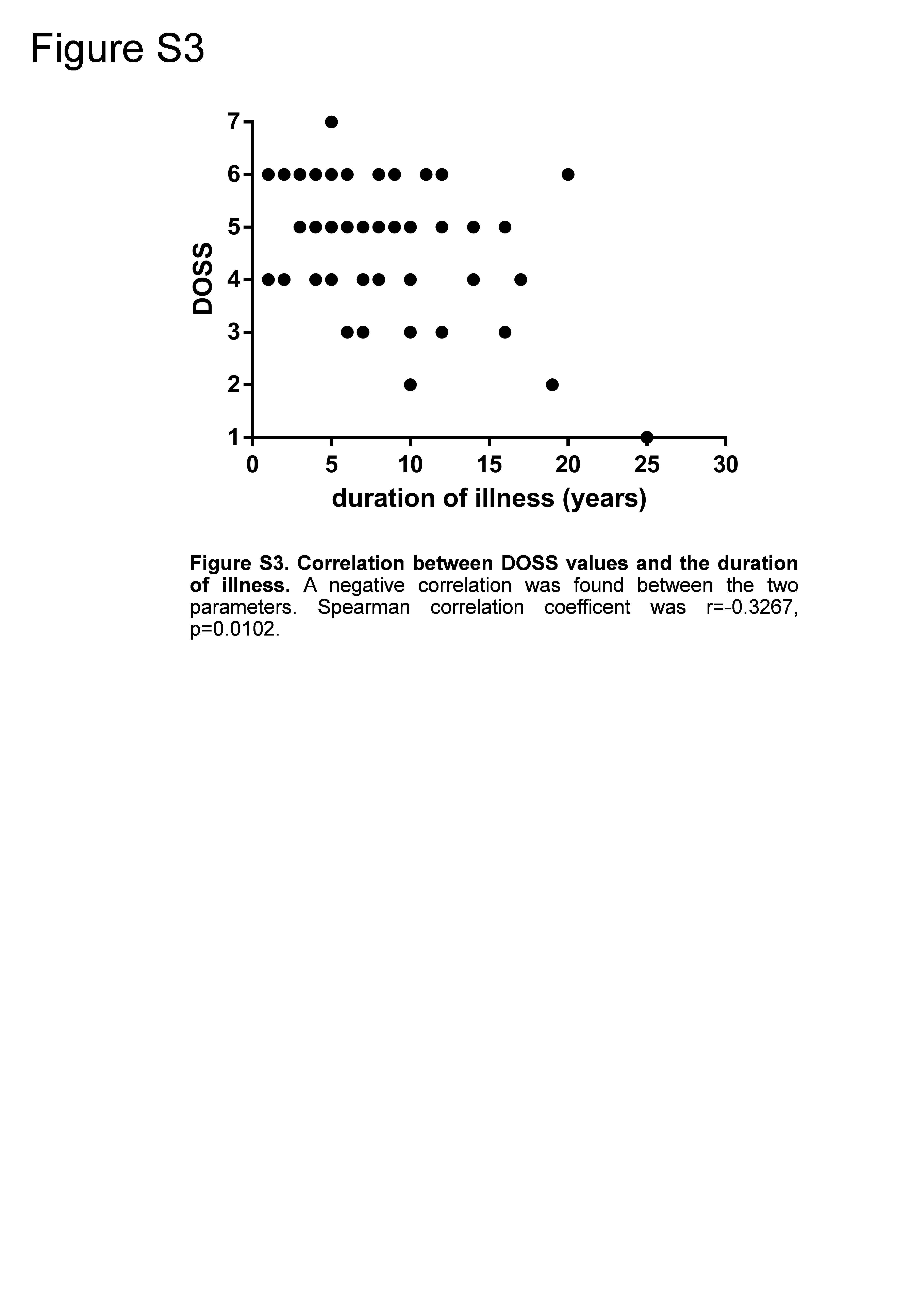

Supplement: Supplementary file 3 — Supplementary Figure 3. [file 41598_2020_72250_MOESM3_ESM.tif]

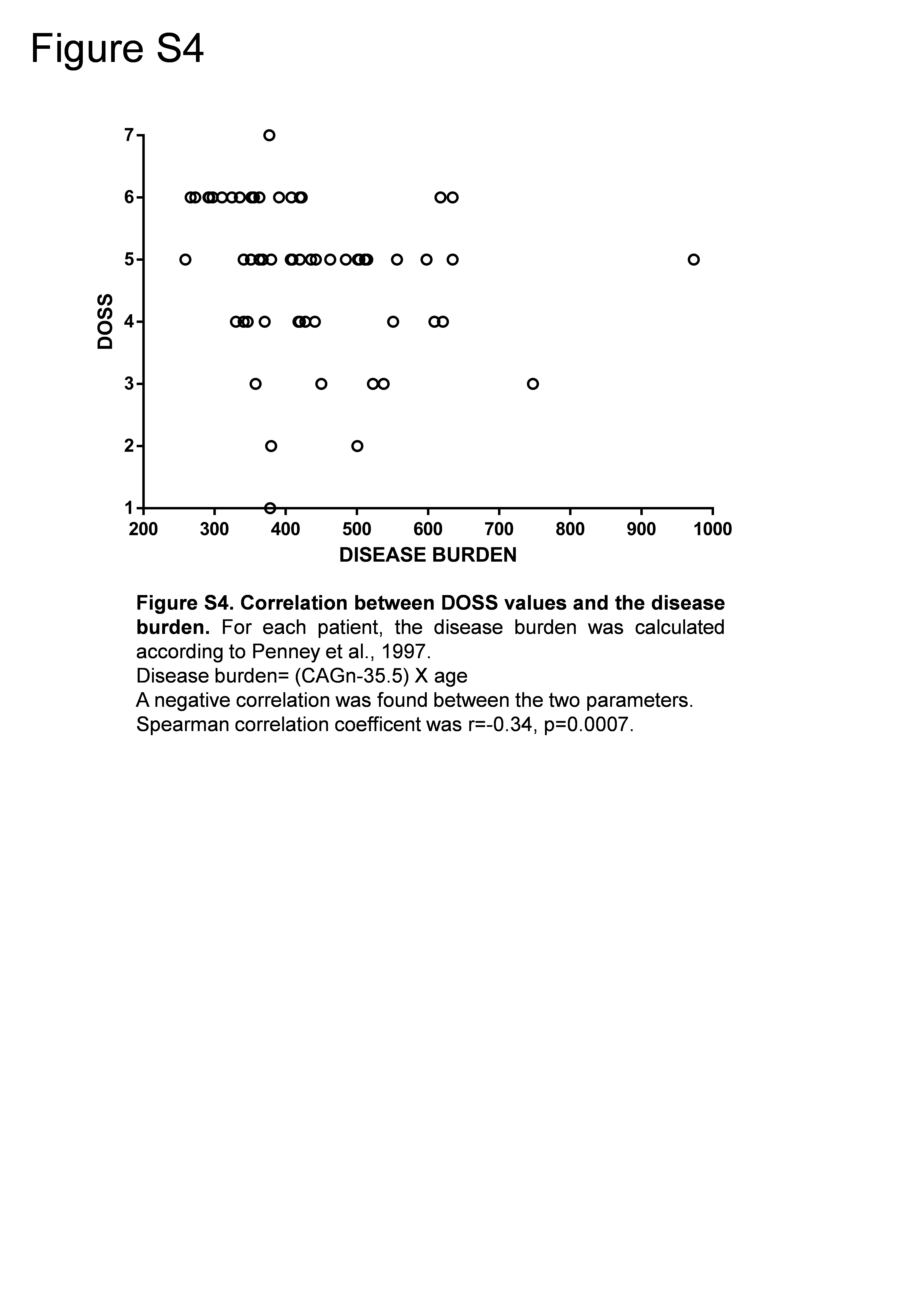

Supplement: Supplementary file 4 — Supplementary Figure 4. [file 41598_2020_72250_MOESM4_ESM.tif]

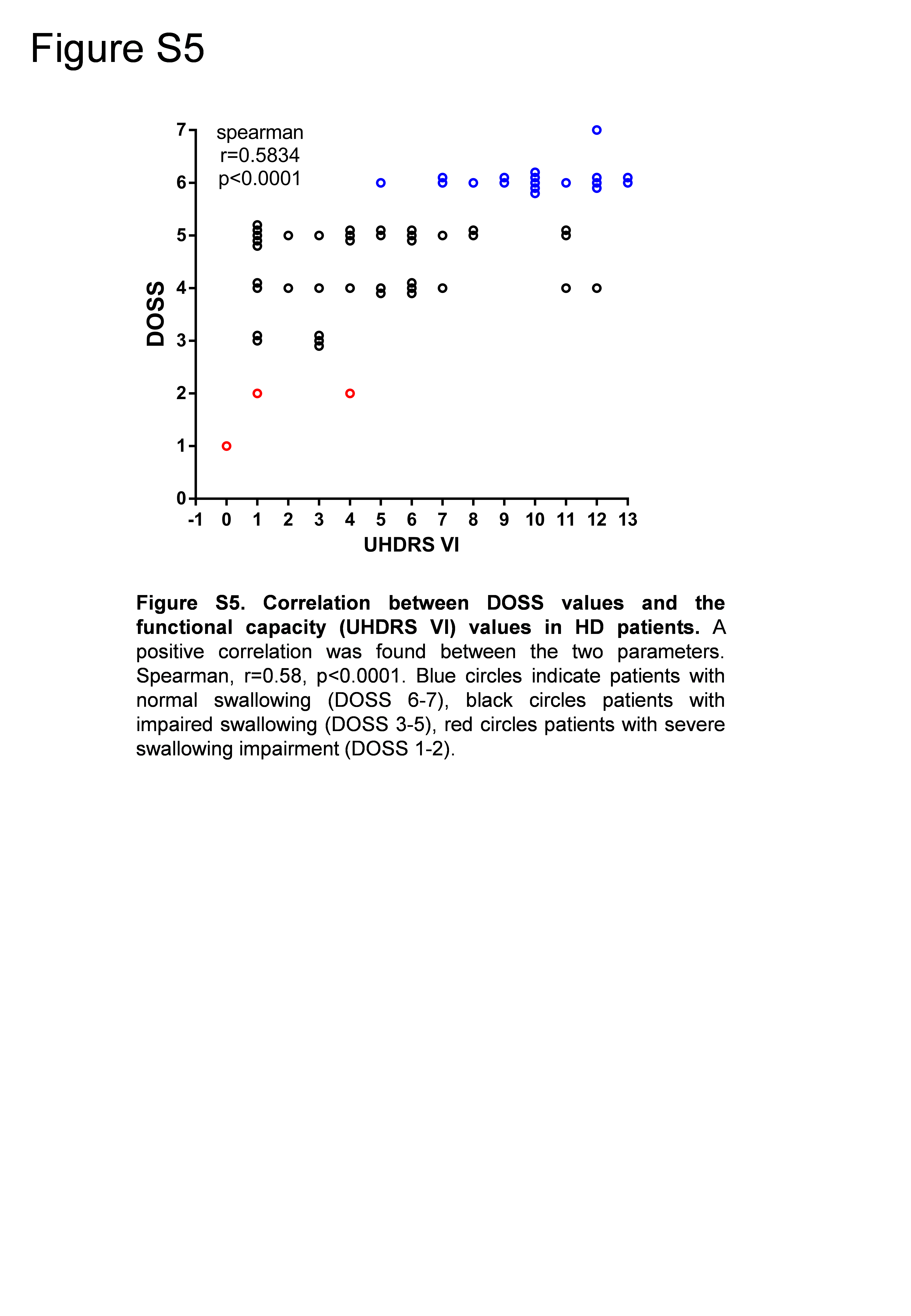

Supplement: Supplementary file 5 — Supplementary Figure 5 [file 41598_2020_72250_MOESM5_ESM.tif]

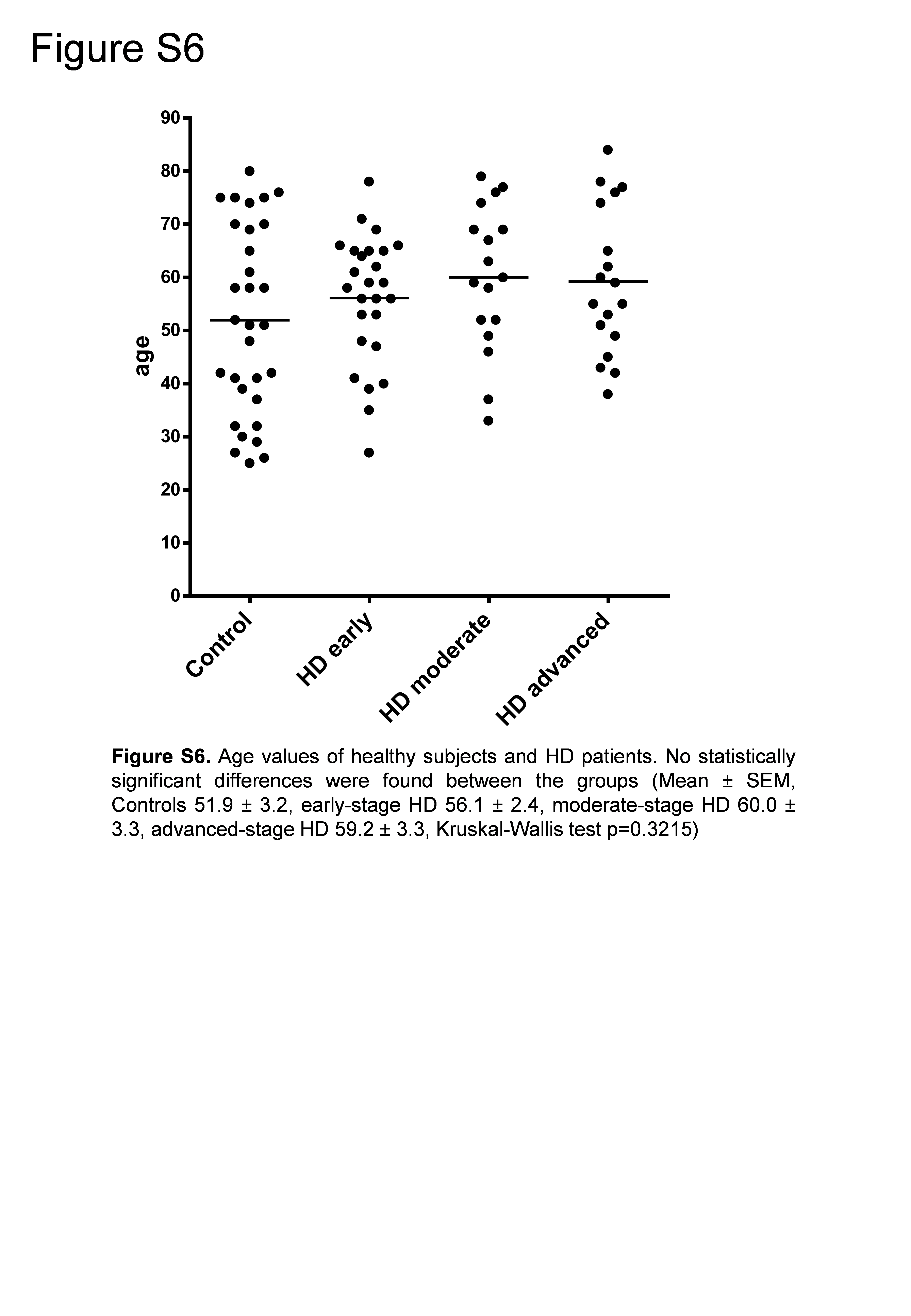

Supplement: Supplementary file 6 — Supplementary Figure 6. [file 41598_2020_72250_MOESM6_ESM.tif]
