# Supplementary material for: Fiberoptic endoscopic evaluation of swallowing in early-to-advanced stage Huntington’s disease
Source: Sci Rep. 2020 Sep 17;10:15242. doi: 10.1038/s41598-020-72250-w (PMC7499207; doi:10.1038/s41598-020-72250-w)
Supplement: Supplementary file 7 — Supplementary Table 1. [file 41598_2020_72250_MOESM7_ESM.docx]

|  | **Controls** | | **HD patients** | | | | | | | |
| --- | --- | --- | --- | --- | --- | --- | --- | --- | --- | --- |
|  | **n=31** | | **all patients n=61** | | **early-stage n=26** | | **moderate-stage n=17** | | **advanced-stage n=18** | |
| **Characteristic** | **Mean ± SEM** | **Range** | **Mean ± SEM** | **Range** | **Mean ± SEM** | **Range** | **Mean ± SEM** | **Range** | **Mean ± SEM** | **Range** |
| **Sex (M/F)** | 16/15 |  | 27/34 |  | 10/16 |  | 8/9 |  | 9/9 |  |
| **Age (years)** | 51.9 ± 3.2 | 25-80 | 58.1 ± 1.7 | 27-84 | 56.1 ± 2.4 | 27-78 | 60.0 ± 3.3 | 33-79 | 59.2 ± 3.3 | 38-84 |
| **CAG repeats on upper allele** |  |  | 43.6 ± 0.5 | 39-59 | 43.2 ± 0.8 | 39-59 | 43.8 ± 0.8 | 40-51 | 44.1 ± 0.8 | 39-52 |
| **Onset (years)** |  |  | 50.4 ± 1.6 | 23-76 | 50.2 ± 2.2 | 23-73 | 53.4 ± 3.2 | 30-76 | 47.9 ± 3.0 | 24-69 |
| **Duration of illness (years)** |  |  | 8.1 ± 0.6 | 1-25 | 6.3 ± 0.8 | 1-20 | 6.6 ± 0.7 | 3-12 | 11.9 ± 1.2 | 5-25 |
| **UHDRS I** |  |  | 49.3 ± 3.1 | 7-106 | 30.2 ± 2.3 | 7-52 | 47.8 ± 3.8 | 27-92 | 78.2 ± 3.6 | 52-106 |
| **UHDRS II** |  |  | 126.5 ± 10.6 | 0-331 | 161.0 ± 12.7 | 86-331 | 91.5 ± 10.0 | 6-150 | 25 ± 25 | 0-75 |
| **UHDRS III** |  |  | 22.2 ± 2.5 | 2-44 | 21.6 ± 4.5 | 2-44 | 22.7 ± 4.8 | 11-44 | 22.4 ± 4.1 | 6-38 |
| **UHDRS IV** |  |  | 13.4 ± 0.9 | 1-25 | 19.4 ± 0.8 | 12-25 | 11.2 ± 0.6 | 7-15 | 4.9 ± 0.8 | 1-9 |
| **UHDRS V** |  |  | 67.5 ± 2.2 | 30-100 | 79.8 ± 1.9 | 70-100 | 65.3 ± 2.3 | 50-80 | 47.3 ± 2.4 | 30-60 |
| **UHDRS VI** |  |  | 6.2 ± 0.5 | 0-13 | 10.1 ± 0.4 | 7-13 | 5.1 ± 0.2 | 4-6 | 1.6 ± 0.2 | 0-3 |

**Supplementary Table S1.** Demographic, genetic, and clinical data of all the subjects enrolled in the study.

**Fiberoptic endoscopic evaluation of swallowing in early-to-advanced stage Huntington’s disease**

Antonio Schindler, Nicole Pizzorni, Jenny Sassone, Lorenzo Nanetti, Anna Castaldo, Barbara Poletti, Federica Solca, Francesca Pirola, Laura Lazzari, Marco Stramba-Badiale, Agnese Rossi, Vincenzo Silani, Caterina Mariotti, and Andrea Ciammola
